# Supplementary material for: Gene Therapy in Combination with Nitrogen Scavenger Pretreatment Corrects Biochemical and Behavioral Abnormalities of Infant Citrullinemia Type 1 Mice
Source: Int J Mol Sci. 2022 Nov 29;23(23):14940. doi: 10.3390/ijms232314940 (PMC9736988; doi:10.3390/ijms232314940)
Supplement: Supplementary file 1 [file ijms-23-14940-s001.zip › ijms-2045759-supplementary.pdf]

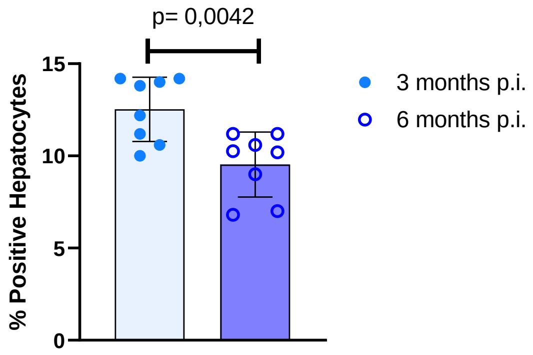

Supplementary Figure S1. Quantification of the percentage of cells containing the AAV genome in *Ass1^{fold}* mice by ISH. *Ass1^{fold}* mice pretreated with SOC and administered with the VTX-804 vector were sacrificed three or six months later and AAV genomes were visualized in liver sections by ISH using a specific probe for the EAlbAAT promoter present in the expression cassette. Percentage of cells containing the viral genome were quantified by analyzing a minimum of 4000 cells/mouse. Data are represented as mean ± SEM and Student *t* test was performed.

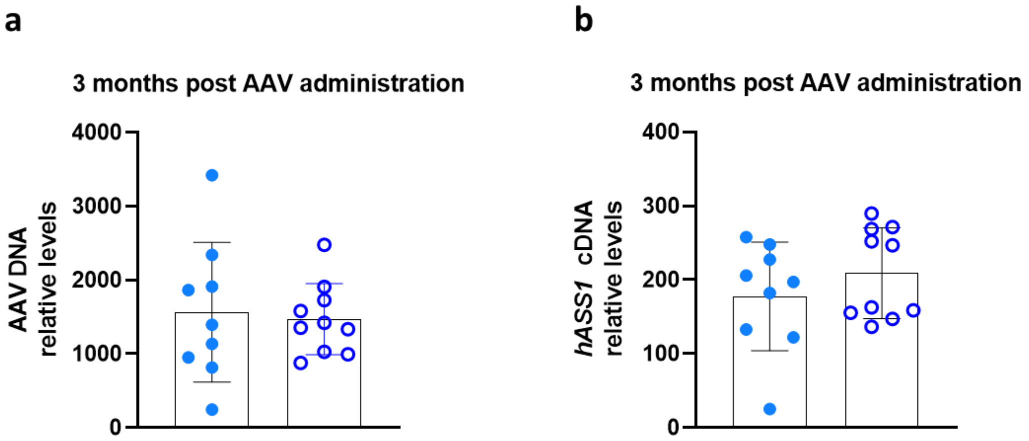

Supplementary Figure S2. SOC does not interfere with AAV transduction or expression in wild-type mice. C57BL/6 wild type mice pretreated or not with SOC were sacrificed at three months of age for the analysis of AAV transduction (a) and transgene expression (b) by qPCR and qRT-PCR respectively. Total DNA values were normalized against *Gapdh* levels (*10E05), total RNA levels against histone (*100). Data are represented as mean ± SEM. (C57BL/6 without SOC, closed circles, n=9; C57BL/6 with SOC, open circles, n=10). No statistically significant differences were observed (Student *t* test).

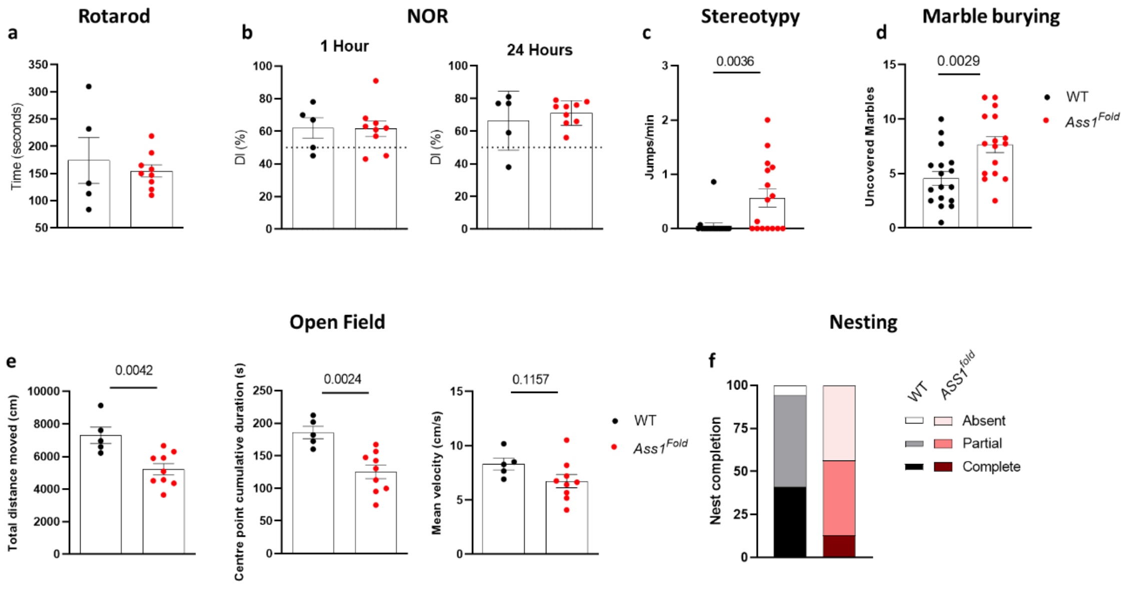

Supplementary Figure S3. Behavioral characterization of WT and *Ass1^{fold}* mice at two months of age. Two-month-old *Ass1^{fold}* mice were subjected to several tests to determine potential deficiencies in behavior. Rotarod test to assess motor coordination (a), NOR test to evaluate memory (b), open field test for locomotor activity and anxiety (e), hopping stereotypy (c) and marble burying (d) and nest building (f) tests to study innate behaviors.

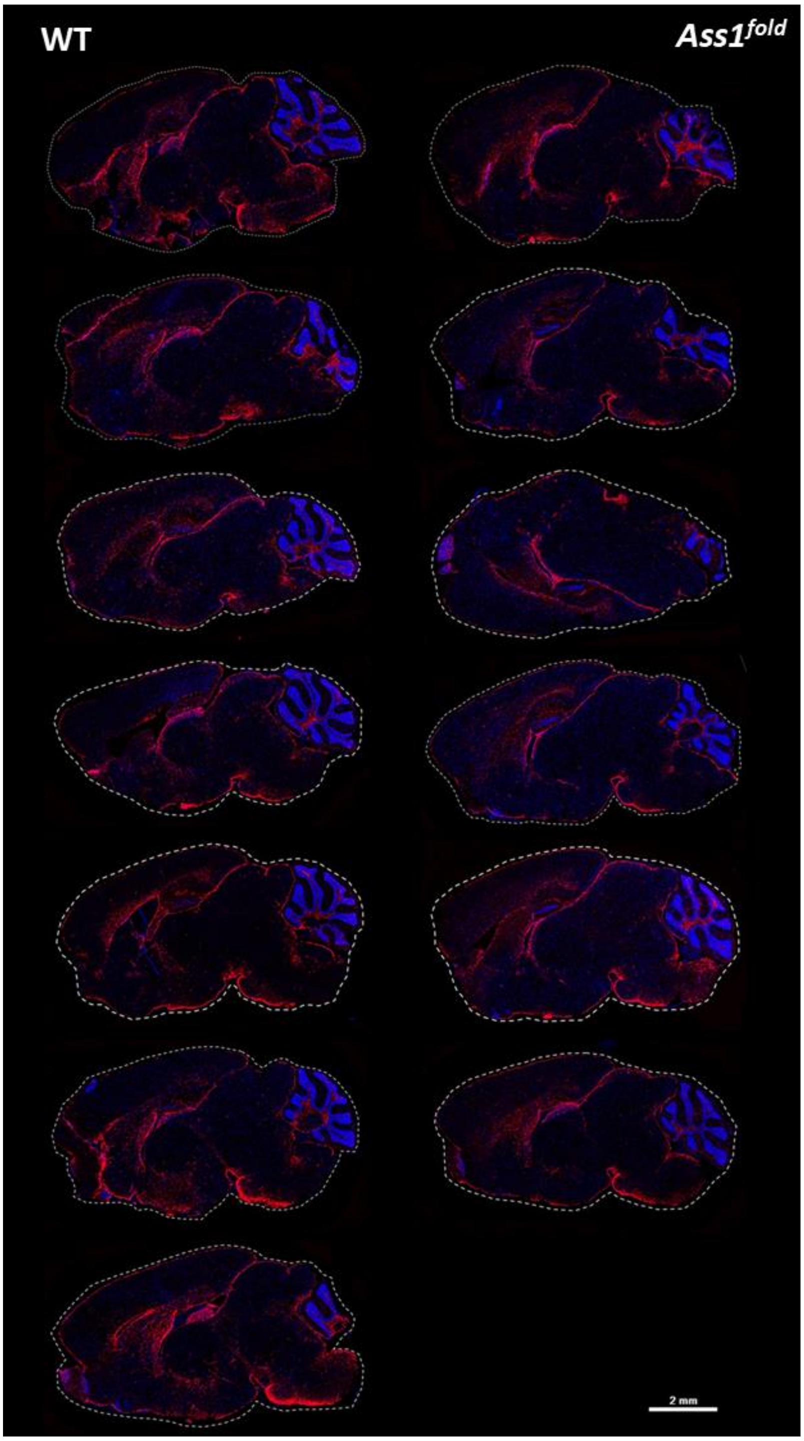

Supplementary Figure S4. Spatial pattern of GFAP expression in brain samples of WT and *Ass1*^{fold} mice. Mice with the indicated genotypes were sacrificed at three months of age for immunohistological analysis of GFAP expression (red). Images were acquired with a Vectra® Polaris™ scanner (n=13). The structure of the brain is delimited by dotted lines. Cell nucleus are in blue color.

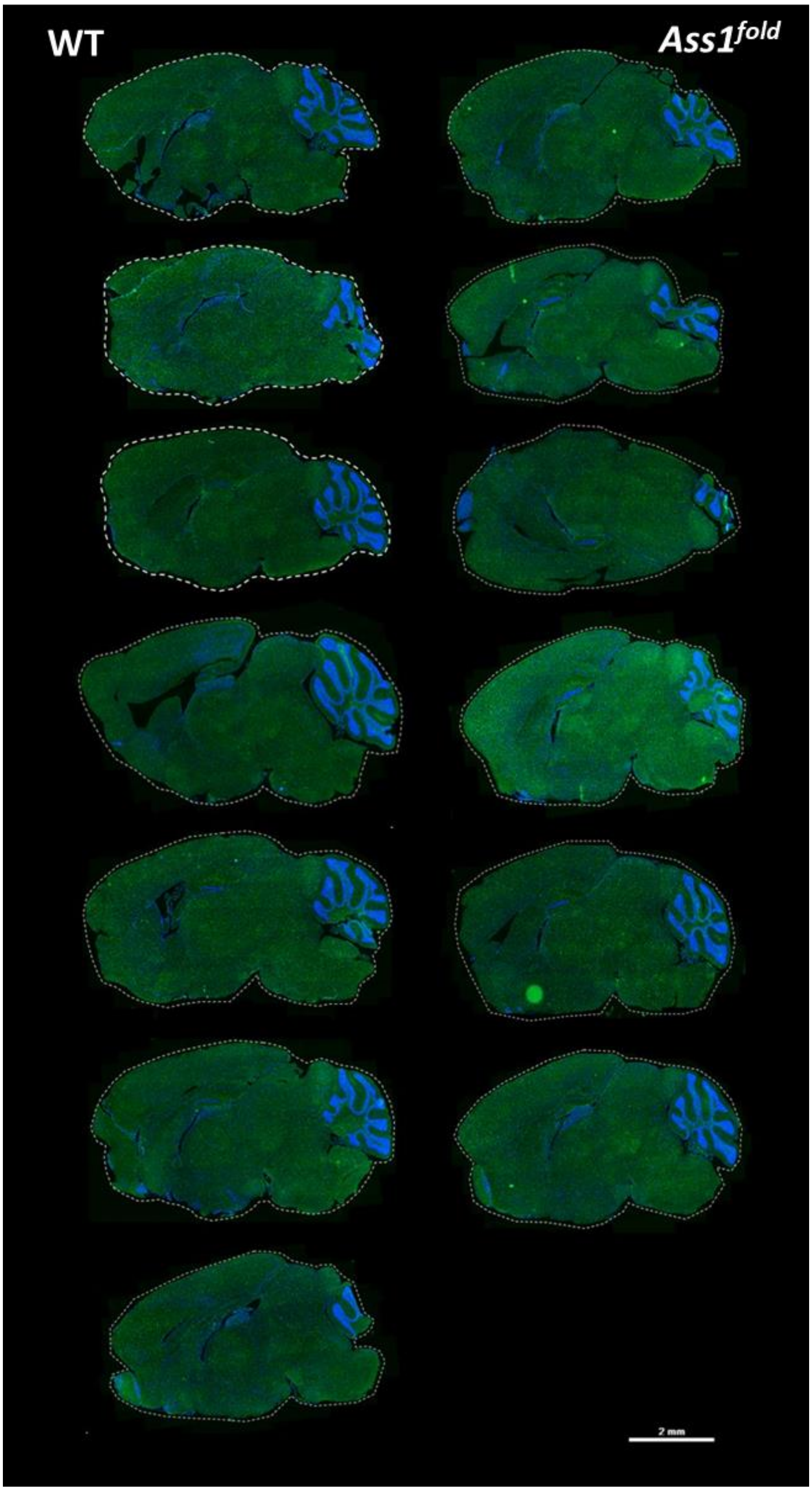

Supplementary Figure S5. Detection of microglia activation by immunofluorescence. Mice with the indicated genotypes were sacrificed at three months of age for immunohistological analysis of IBA1 expression (green). Images were acquired with a Vectra® Polaris™ scanner (n=13). The structure of the brain is delimited by dotted lines.

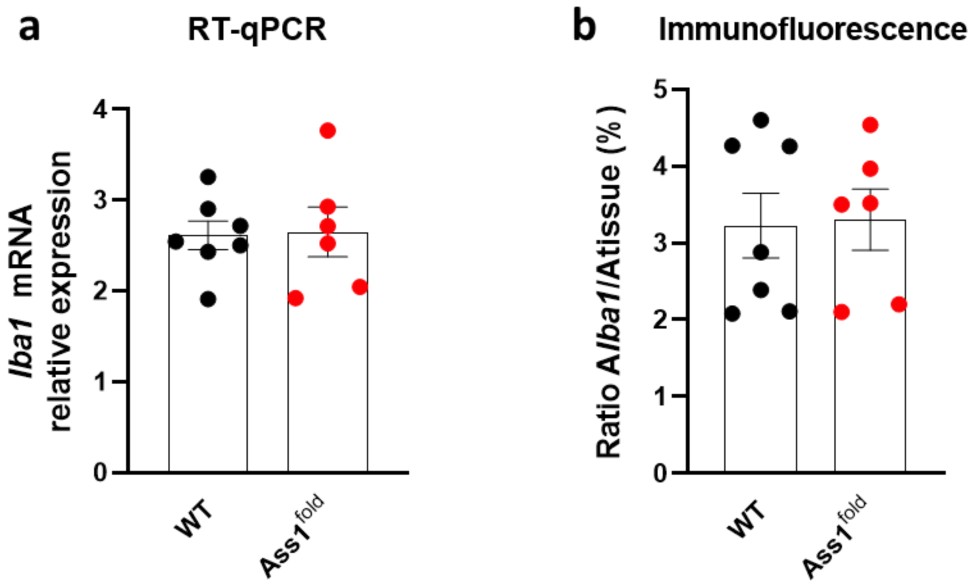

Supplementary Figure S6. Total *Iba1* expression is similar in WT and *Ass1*^{fold} mice. Mice with the indicated genotypes were sacrificed at three months of age for analysis of *Iba1* expression. Quantification of *Iba1* expression by RT-qPCR and normalized against histone (*100) (a) and by immunofluorescence showing the ratio of IBA1 positive are respect tissue area (%). *Iba1* was quantified automatically using Fiji – ImageJ Software. Data are represented as mean ± SEM. (WT n=7; *Ass1*^{fold} n=6). No statistically significant differences were observed (Student *t* test).
